# Experimental results on nonlinear distortion compensation using photonic reservoir computing with a single set of weights for different wavelengths

**DOI:** 10.1038/s41598-023-48816-9

**Published:** 2023-12-04

**Authors:** Emmanuel Gooskens, Stijn Sackesyn, Joni Dambre, Peter Bienstman

**Affiliations:** 1 Photonics Research Group, Department of Information Technology, Ghent University - imec, Ghent, Belgium; 2 Center for Nano-and Biophotonics (NB-Photonics), Ghent University, Ghent, Belgium; 3 IDLab, Department of Electronics and Information Systems, Ghent University - imec, Ghent, Belgium

**Keywords:** Mathematics and computing, Optics and photonics

## Abstract

Photonics-based computing approaches in combination with wavelength division multiplexing offer a potential solution to modern data and bandwidth needs. This paper experimentally takes an important step towards wavelength division multiplexing in an integrated waveguide-based photonic reservoir computing platform by using a single set of readout weights for up to at least 3 ITU-T channels to efficiently scale the data bandwidth when processing a nonlinear signal equalization task on a 28 Gbps modulated on-off keying signal. Using multiple-wavelength training, we obtain bit error rates well below that of the $1.5 \times \,10^{-2}$ forward error correction limit at high fiber input powers of 18 dBm, which result in high nonlinear distortion. The results of the reservoir chip are compared to a tapped delay line filter and clearly show that the system performs nonlinear equalization. This was achieved using only limited post processing which in future work can be implemented in optical hardware as well.

## Introduction

The need for ever higher data bandwidths in all aspects of the current digital society (video streaming, cloud services,...) requires ever improved data processing in terms of data bandwidth and energy consumption^1^. Between 2018 and 2022, global data bandwidth nearly tripled with an increase of 28% in 2022 alone, reaching an estimated total by the end of 2022 just shy of 1 Pbps^2^. Traditional electronics-based systems are reaching physical limits due to transistor size limitations and charging of electrical lines^3^. Photonics-based hardware approaches offer a number of advantages, in particular high data bandwidth. This is especially so when enhanced through wavelength division multiplexing (WDM), which makes them attractive alternatives. There are many potential photonics-based hardware implementations for data processing, such as those focusing on matrix multiplication which is of interest for convolutional processing and deep learning applications^4–6^. In this work however, we are concerned with reservoir computing systems^7–9^. Reservoir computing refers to using a randomly initialised dynamical system, called the reservoir, which processes the information. This system is left untrained, but a simple linear readout layer is added to it. Only this linear readout is trained, leading to a simple training scheme. This, combined with flexibility and insensitivity to manufacturing errors, makes reservoir computing well suited for a photonic-based hardware implementation. Examples of such photonic reservoir computing (PRC) systems are approaches consisting of a single non-linear node with feedback and free-space reservoir systems^10–28^. This work however focuses on a spatially extended implementation with multiple nodes, fabricated in silicon photonics. Compared to free-space or fiber-based approaches, this benefits system compactness and stability. In^29,30^ such integrated PRC chips were shown to be able to perform arbitrary boolean logic operations with memory, header recognition and signal equalization.

This work will also target the above-mentioned signal equalization task, which aims to eliminate the (nonlinear) distortion of an optical signal after propagation through an optical link. One of the fundamental problems at the high bandwidths that are being targeted in this work, is the Kerr effect in optical fibers. Especially at high powers, it can be responsible for multiple nonlinear optical effects, like self-phase modulation (SPM)^31^. Signal equalization is currently typically handled by electronic Digital Signal Processing (DSP) chips, but these struggle at ever increasing data bandwidths. The capability of PRC to eliminate nonlinear distortion through fast in-hardware photonic processing was already demonstrated in^30,32^. In^33^ it was shown using simulations how the wavelength dimension in waveguide-based photonic reservoir computing could be exploited in order to enhance the footprint-to-bandwidth ratio. Here, we extend that theoretical work by experimentally verifying its results using a chip, measurement setup and methods similar to those used in^29,30^.

The remainder of this paper is structured as follows. First, a short overview is given on the photonic reservoir used. In the next section, the methods used for obtaining the necessary data and the processing thereof are discussed in detail. Then, we present and discuss the results obtained from the process, before concluding in a final section.

### Photonic reservoir design

The design of this photonic reservoir is based on the four-port architecture, which is a power efficient evolution of the swirl architecture^34^, by replacing all three-ports by four-ports^30,35^. The chip architecture causes interference between the different light paths reaching each reservoir node. This can be mathematically modelled by representing the optical signals propagating through the reservoir by complex numbers, i.e. containing both amplitude and phase information. To fully describe the reservoir dynamics we performed optical circuit simulations, using Photontorch as in^33^. The node signals are then linearly combined with trained weights in order to solve a certain task. The node signals can be either converted by the quadratic photodetector nonlinearity before a linear combination (the so-called electrical readout), or after a linear combination using complex-valued weights (the so-called optical readout). The nodes are implemented as 3 × 3 multimode interferometers (MMIs), which distribute the optical power in their input ports equally over their output ports.

**Figure 1:**
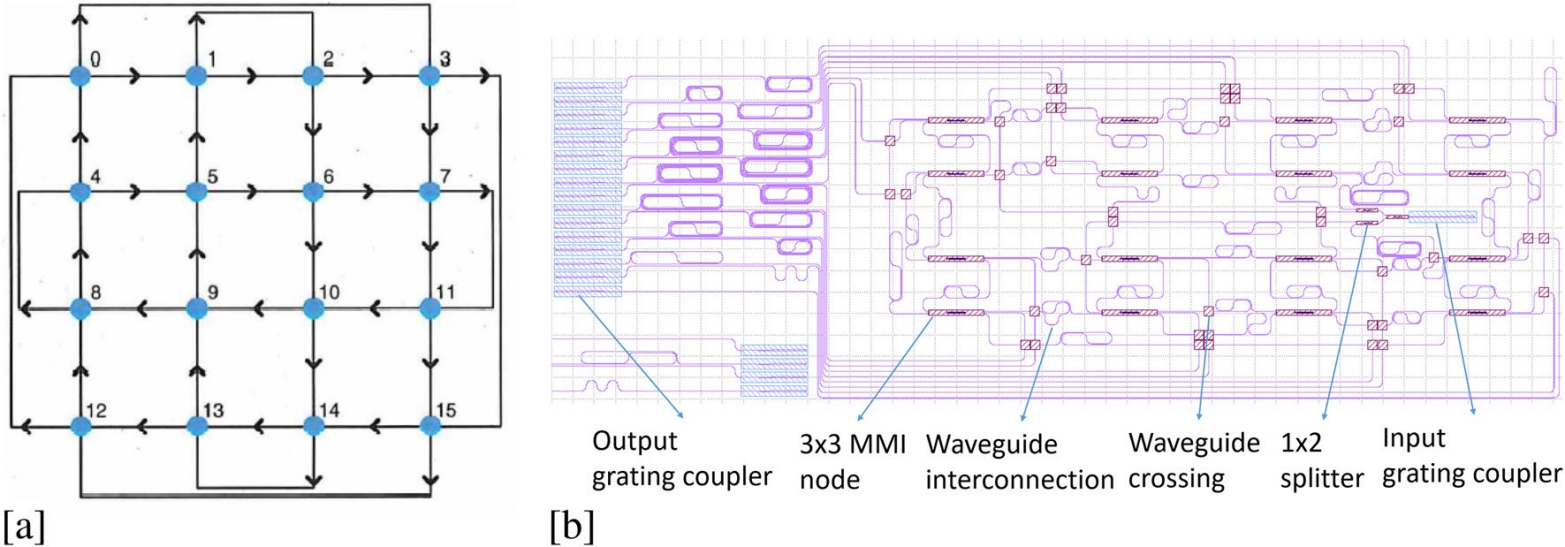
(**a**) Schematic four-port architecture^35^. (**b**) Mask layout of integrated circuit.

Two inputs and two outputs are used for inter-node connections with the remaining input and output serving as interfaces to and from the reservoir. The reservoir studied in this paper has 16 nodes in a 4 by 4 configuration as is shown in Fig.1. The chip is made on a silicon nitride platform (through Ligentec), and has a footprint of $\approx 31 \; {\text {mm}}^{2}$. The interconnection length between nodes was $\approx$ 2.1 mm for the inner reservoir connections, $\approx$ 4.2 mm for the long vertical outer connections and $\approx$ 6.3 mm for the long horizontal outer node connections. The interconnection loss was $\approx$ 0.47 dB/cm.

### Measurement setup and post processing

**Figure 2:**
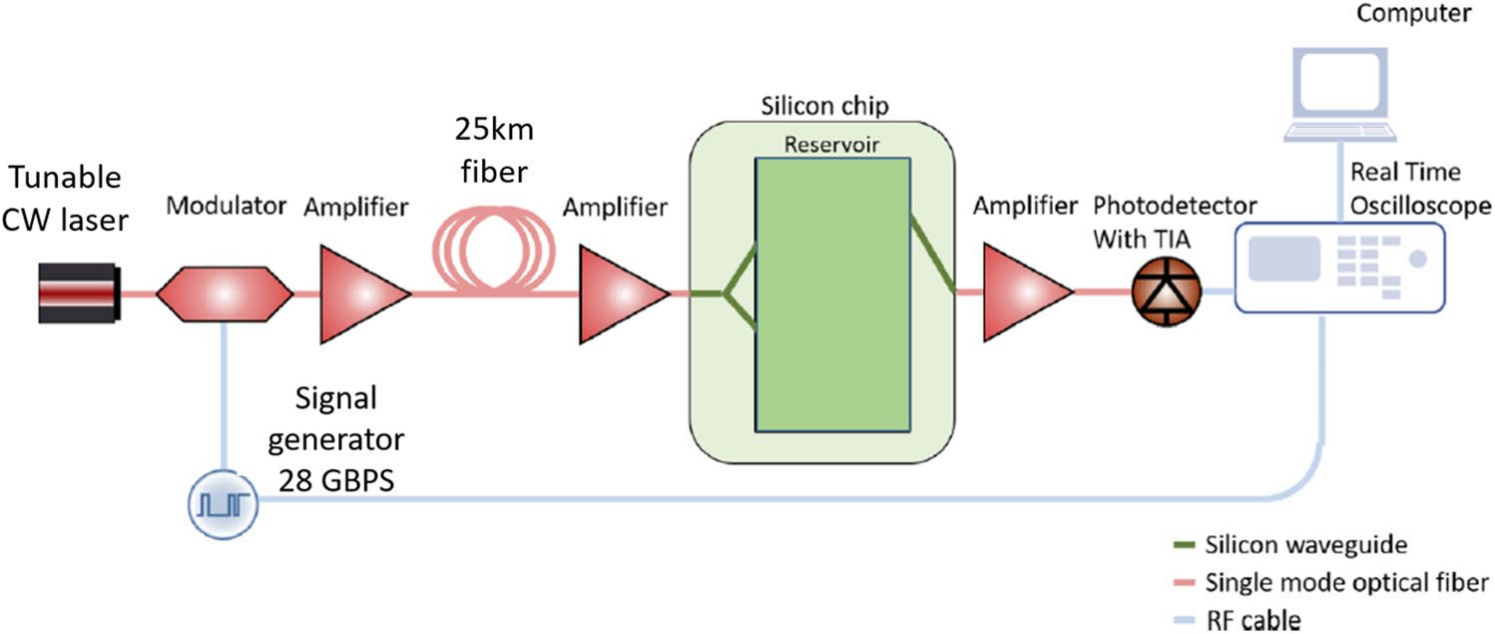
Schematic illustration of the setup used in the experiment^30^.

**Figure 3:**
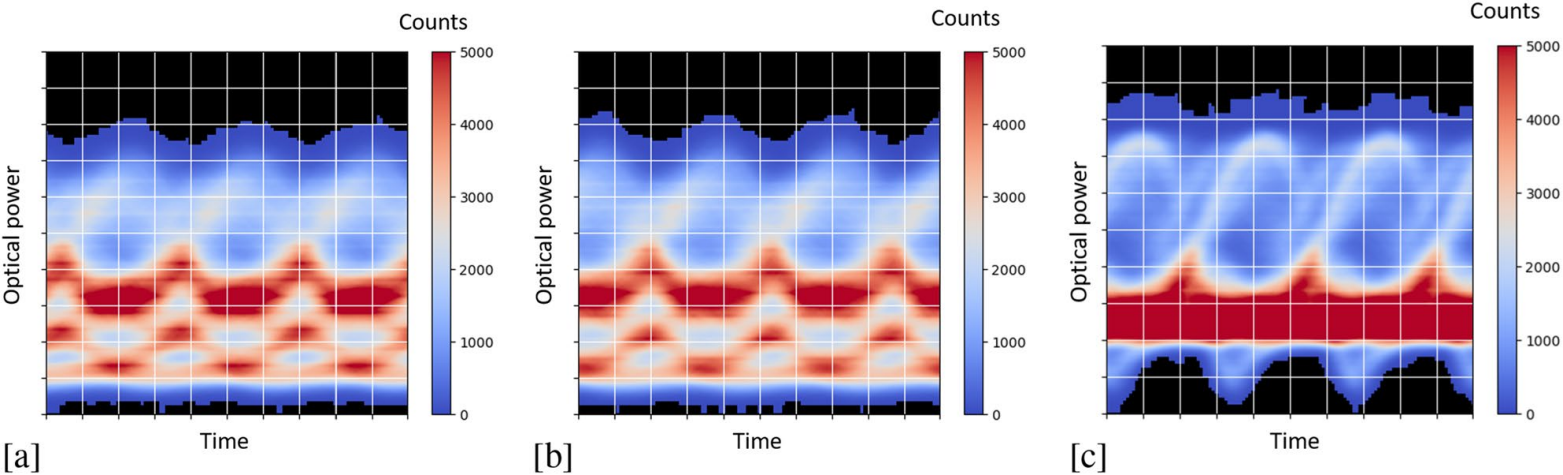
Eye diagrams at the output of the fiber for different input powers. (**a**) 5 dbm (**b**) 10 dbm (**c**) 18 dbm.

The experimental setup is shown in Fig. 2. A single-mode fiber of 25 km with an attenuation of 0.2 dB/km at 1550 nm was added between modulator and chip in order to provide signal distortion for the signal equalization task. The signal is amplified before being sent through the fiber in order to increase nonlinear distortion effects. By setting the fiber input power, we can specify the difficulty of the equalization task to be solved by our reservoir computer, as can be seen from the eye diagrams in Fig.  3.

This paper will study 3 standard ITU-T 50 GHz separated wavelengths namely [1552.122, 1552.524, 1552.926] nm. It is important to mention that the experiments do not use a true WDM signal, where all wavelengths are injected simultaneously into the fiber, but make use of wavelength sweeps over the WDM wavelengths. Although this neglects nonlinear crosstalk between the wavelengths, it is an important first step to show that the same weights can be still be used in a situation like this.

An amplifier before the chip is set in such a way that the optical power at the input of the reservoir is the same for all experiments. This minimises signal-to-noise variations for different fiber input powers, and ensures a fair comparison.

**Figure 4:**
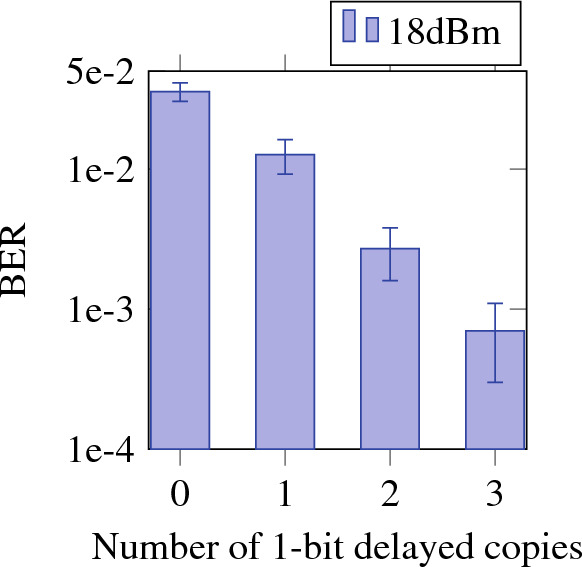
Average BER over the cross validation iterations as a function of number of delayed copies. Error bars indicate the standard deviation of the BER over the cross validation iterations. The fiber input power was 18 dBm and all 3 ITU-T wavelengths were included in training.

The experiments presented in this paper are all done using a so-called electrical readout strategy. This means that the nodes are optically probed and detected by a photodiode, one at a time. The entire input signal, modulated using on-off keying at 28 Gbps, is repeated in time in order to accomplish this. The electrical time traces collected at each photodiode are saved on a computer and the linear combination of these traces is done in post-processing on a computer. This is in contrast to a so-called optical readout strategy, where the optical signals are weighted and combined on-chip in the analogue optical domain, before being sent to a single photodiode. This latter method is certainly feasible, but has a higher technological complexity, which brings more challenges. The optical readout is subject to ongoing experiments and out of the scope of this paper. Long-term however, such a strategy is the way to go^30^. For such an optical readout strategy to work efficiently with WDM in the future, we now use only a single set of weights for all sequentially-measured wavelengths. Indeed, having a separate sets of weights for every wavelength would significantly increase chip footprint, electrical steering complexity and power consumption, thereby negating the aim of this work. Even though for the electrical readout strategy employed here, these problems are less present, we will still limit the readout to one set of weights for all wavelengths, in order to showcase the viability of the long term strategy. Obviously, having only a single set of weights means that only the same task can be executed for the different wavelengths. High chip losses, due to suboptimal design and fabrication of the MMIs, necessitate the use of another amplifier after the chip in order to amplify the signal to within the detection range of the photodetector. Finally, the signal is saved electronically and post-processed on a computer. Since we do not measure different channels at the same time, but rather sequentially, we temporally realign the channels by optimising the time offset for every node using a Gaussian optimisation algorithm^36^, which minimizes the average bit error rate (BER) across the used wavelength channels for a validation dataset. The training of the weights consists of a ridge regression algorithm, as implemented by the sklearn Python library in which normalization is allowed, and makes use of a cross validation implementation. Indeed, the data for each wavelength is split into 10 folds, and in each iteration 9 folds serve as training data for the ridge regression and 1 fold serves as test data. In this way, after 10 iterations, all 10 folds were used for testing. The average and standard deviation of the BERs over the 10 iterations are then used to display the results. (The use of ridge regression instead of the more complicated Adam gradient descent algorithm as in^33^ was possible in case of the electrical readout only having real-valued weights, versus the complex-valued weights of an optical readout.) The labels used for training are the symbols in the digital on-off keying bit stream. The cross validation data set consisted of 20 000 bits. For the optimization of the time offsets, a separate validation set consisting of 10 000 bits was used. Note that when eventually moving from electronic weights to non-volatile optical weights, these could have a much lower resolution. However, the effects of this limited weight resolution can be counteracted using specific training algorithms^37^. During this postprocessing, three additional 1-bit delayed copies of the node signals are used in conjunction with the original measured node signal. This increases the number of effective outputs of the reservoir, and is similar to adding a feedforward equalizer in the electrical domain on top of the optical reservoir. In Fig. 4 it is shown how adding of these delayed copies favorably impacts performance. Although each delayed copy has its own readout weight, we still opted to make these weights independent on wavelength, to align closer to a future optical readout implementation.

## Results

**Figure 5:**
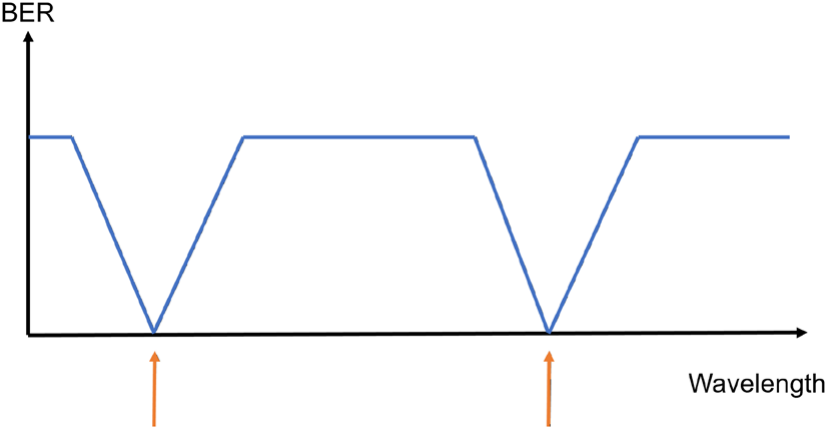
When training for multiple wavelengths good performance is expected at those wavelengths, and a limited wavelength range centered around these^33^.

The main reason why reservoir performance normally goes down when varying the wavelength/frequency is the resulting variation in phase shifts in the waveguide interconnections. This leads to altered signal mixing for which the readout was not trained and the time offsets were not optimized, leading to an incorrect weighting and recombination of node outputs. However, taking into account more than one wavelength during training allows the readout to adapt to the phase dynamics associated with each wavelength but poses a greater challenge as a machine learning task^33^ (Fig. 5).

**Figure 6:**
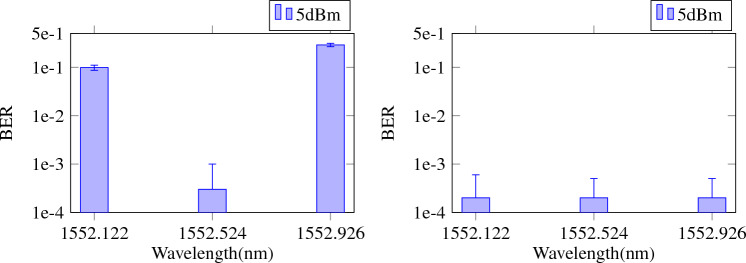
Average BER, with error bars indicating standard deviation over the cross validation iterations for single-wavelength training (left) versus multiple-wavelength training (right) at 5 dBm fiber input power. In the top case the wavelength for which time offsets were optimized and readout weights were trained was 1552.524 nm, whereas in the bottom case, all wavelengths were taken into account.

Figure 6 shows how the performance over the wavelengths in the case of only one wavelength being trained and in the case of multiple wavelengths being trained to show the effect of multiple wavelength training. In the left figure, it is clear that the performance is only good for the wavelength that was trained (1552.524 nm). For the right figure on the other hand, all wavelengths shown were incorporated in the training set, and this clearly results in better performance over the entire wavelength range. Again, we want to stress that we use the same weights for all the wavelengths in this case.

**Figure 7:**
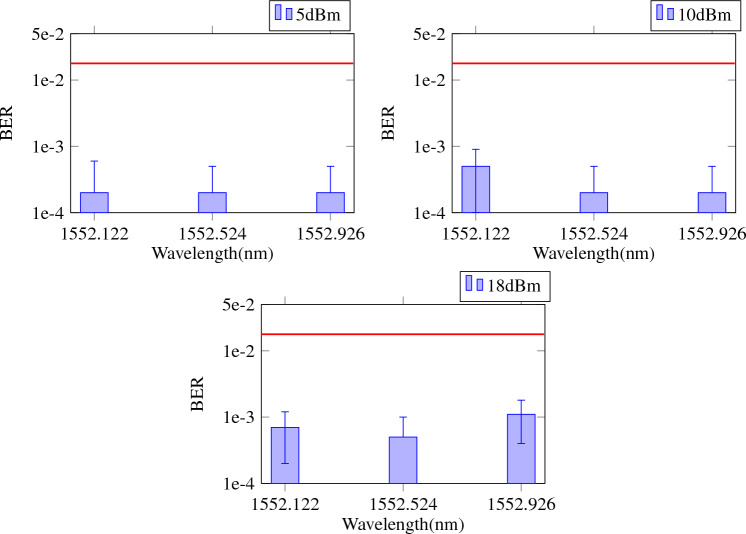
Average BER, with error bars indicating standard deviation over the cross validation iterations for multiple wavelength training. All wavelengths displayed were taken into account for time offset optimization and readout weight training. Fiber input powers were 5 dBm, 10 dBm and 18 dBm. The horizontal red line illustrates the relevant FEC limit.

The results in Fig. 6 were for a low fiber input power of 5 dBm, where we do not expect significant nonlinearity. We will now investigate the performance for higher input powers like 10 and 18 dBm. In Fig. 7, it is shown that the system can still perform well for at least up to 3 standard 50 GHz spaced ITU-T wavelengths at these higher levels of difficulty. (There is no reason to assume 3 channels is the limit, and measuring more wavelength channels is a goal of future work.) For all 3 wavelength channels, the BER stays well below $1.5 \times \, 10^{-2}$, which is the forward error correction (FEC) limit of the P1-PTX-2-100G-WDM optical transport system^38^. This system is suitable as a reference, because it has strong dense WDM capabilities in the C-band and is therefore the type of interface that could be paired with this PRC system. Still, for the most difficult case at 18 dBm, the BER starts to degrade, and it becomes harder for the single set of readout weights to equalise all wavelengths equally.

**Figure 8:**
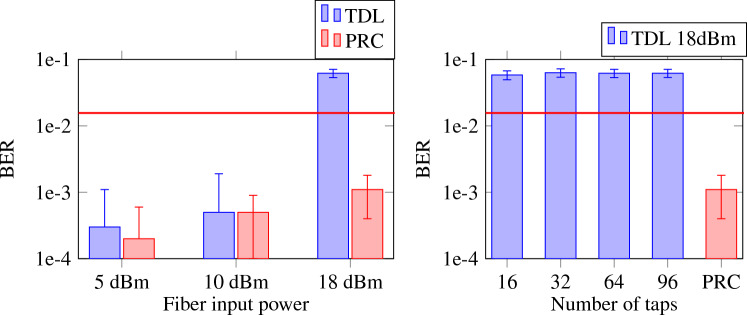
Average BER, with error bars indicating standard deviation, over the cross validation iterations for a tapped delay line, trained and tested for one wavelength (1552.524 nm) in comparison with worst performing single-readout PRC wavelength. (Left) For various levels of input power. (Right) For different number of tapped delay line taps at 18 dBm input power. The horizontal red line illustrates the relevant FEC limit.

Finally, we will compare the performance of our system with an electrical tapped delay line (TDL) filter. The PRC system used 16 nodes with three additional 1-bit delayed copies of the node signals, which translates to 64 readout weights. In order to have a fair comparison, we will also use 64 taps for the TDL. Just as in^30^, the reservoir outperforms such a TDL in the case of significant nonlinear distortion, i.e. when the input power is 18 dBm Fig. 8 (left). This is the case for all wavelengths processed by a single set of weights. Even the worst performing wavelength has a BER well below that of a TDL filter optimised for only one wavelength. Note that the TDL’s inferior performance stems from its inability to equalize the nonlinear distortion (in the reservoir, the combination of interference and a photodiode creates a quadratic nonlinearity). It is also largely independent of the number of taps used, as can be seen in Fig. 8 (right).

## Conclusion

We have experimentally taken a first important step towards WDM in an integrated waveguide-based photonic reservoir computing platform using only limited post processing i.e. a digitally implemented readout and the generating of delayed copies of measured node signals. When using the FEC limit of $1.5 \times \,10^{-2}$ as a benchmark, the single-readout system was shown to work well up to at least 3 standard ITU-T wavelengths for 28 Gbps (nonlinear) signal equalization tasks. The reservoir outperforms a simple tapped delay line filter for higher levels of signal distortion. This confirms the capacity of the system to tackle nonlinear tasks. By reaffirming experimentally that the nonlinear problem solving capability of the system remains intact and that performance does not degrade to unacceptable levels when utilizing a single set of readout weights, integrated waveguide-based photonic reservoir computing platforms are shown to be a potential solution to modern data bandwidth challenges.

## Data Availability

Data underlying the results presented in this paper are available from the corresponding author (emmanuel.gooskens@ugent.be) upon reasonable request.
